# Treatment of retrograde ascending aorta and aortic arch intramural hematoma (IMH) with thoracic endovascular aortic repair (TEVAR)

**DOI:** 10.1186/s13019-025-03653-x

**Published:** 2025-11-11

**Authors:** David Greentree, A. Claire Watkins

**Affiliations:** Department of Cardiothoracic Surgery, Stanford University School of Medicine, Stanford, Palo Alto, CA USA

## Abstract

Typically, the presence of ascending aortic IMH is treated with open surgical repair due to the unpredictability of subsequent rupture. We demonstrate successful endovascular management of retrograde ascending IMH with TEVAR in a 58-year-old, high-risk patient. Assisted by high-quality pre- and intra-operative imaging, TEVAR for type B dissection with retrograde IMH extension into the ascending aorta may offer a less invasive treatment and possibly a better outcome for patients.

## Case report

A 58-year-old female with past medical history of diabetes mellitus, poorly controlled hypertension, end-stage renal disease on hemodialysis and a history of stroke and seizure presented with acute onset midsternal chest pain with radiation to her back and her jaw. Additionally, she had residual lower extremity weakness from a prior stroke and was confined to a wheelchair. The chest pain was stabbing in nature, accompanied by shortness of breath. The patient denied any fever, chills, nausea or vomiting, abdominal pain, or diarrhea. In the ED, her troponin was negative, EKG showed diffuse T wave inversions, transthoracic echocardiogram showed moderate circumferential pericardial effusion.

**Fig. 1:**
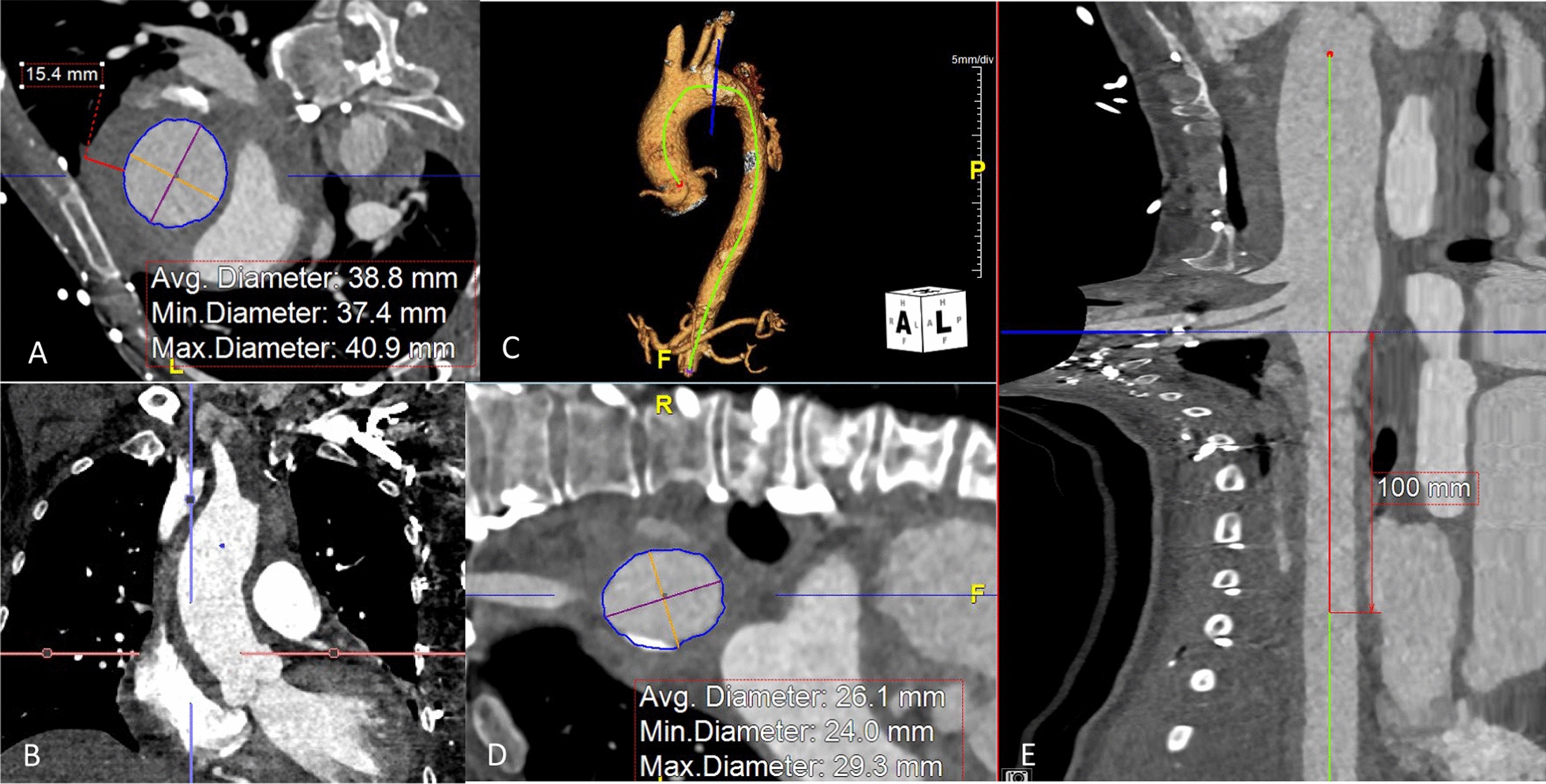
Pre-operative Imaging. **A**, **B** Ascending aorta. **C** 3-D Reconstruction. **D**, **E** Proximal descending aortic landing zone

The patient was hypertensive with a systolic blood pressure of 220 mmHg which was stabilized with labetalol, nitroglycerin and nicardipine. She was tachycardic, with intact neurologic and respiratory status. Computed topographic angiogram of chest demonstrated focal type B dissection with a small primary entry tear in the proximal descending aorta, with retrograde extension of IMH to the aortic root, as well as a small pericardial effusion. Additionally, the right subclavian artery was frankly dissected from the innominate bifurcation to the thoracic inlet. There were small false lumen fenestrations at intercostal origins in the mid-descending aorta, with IMH ending at the level of the superior mesenteric artery. The ascending aorta measured 41 mm mm in its greatest transverse diameter, with and additional 15 mm of IMH. Cardiomegaly and a small pericardial effusion were also noted. Iliofemoral access size could not be verified. (Fig. 1).

**Fig. 2:**
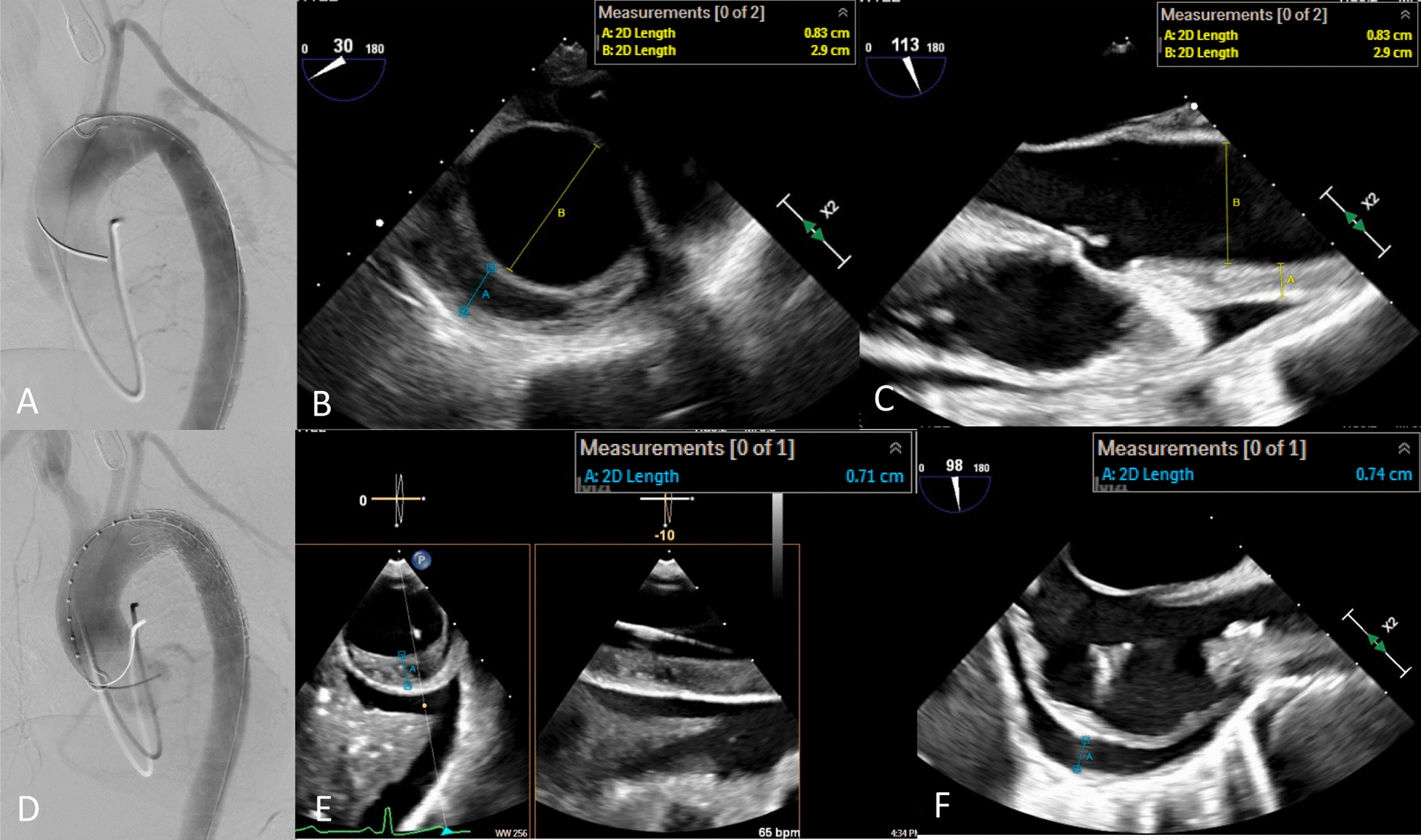
Intraoperative Imaging. **A** Pre-deployment fluoroscopy. **B**, **C** Pre-deployment echo. **D** Post-deployment fluoroscopy. **E** Post-deployment echo. **F** Pre-deployment pericardial effusion on echo

The patient was transferred to a tertiary referral center with a multi-disciplinary team in aortic surgery. Given her risk-profile and the nature of her dissection, she was treated with zone 3 TEVAR (Fig. 2).

**Fig. 3:**
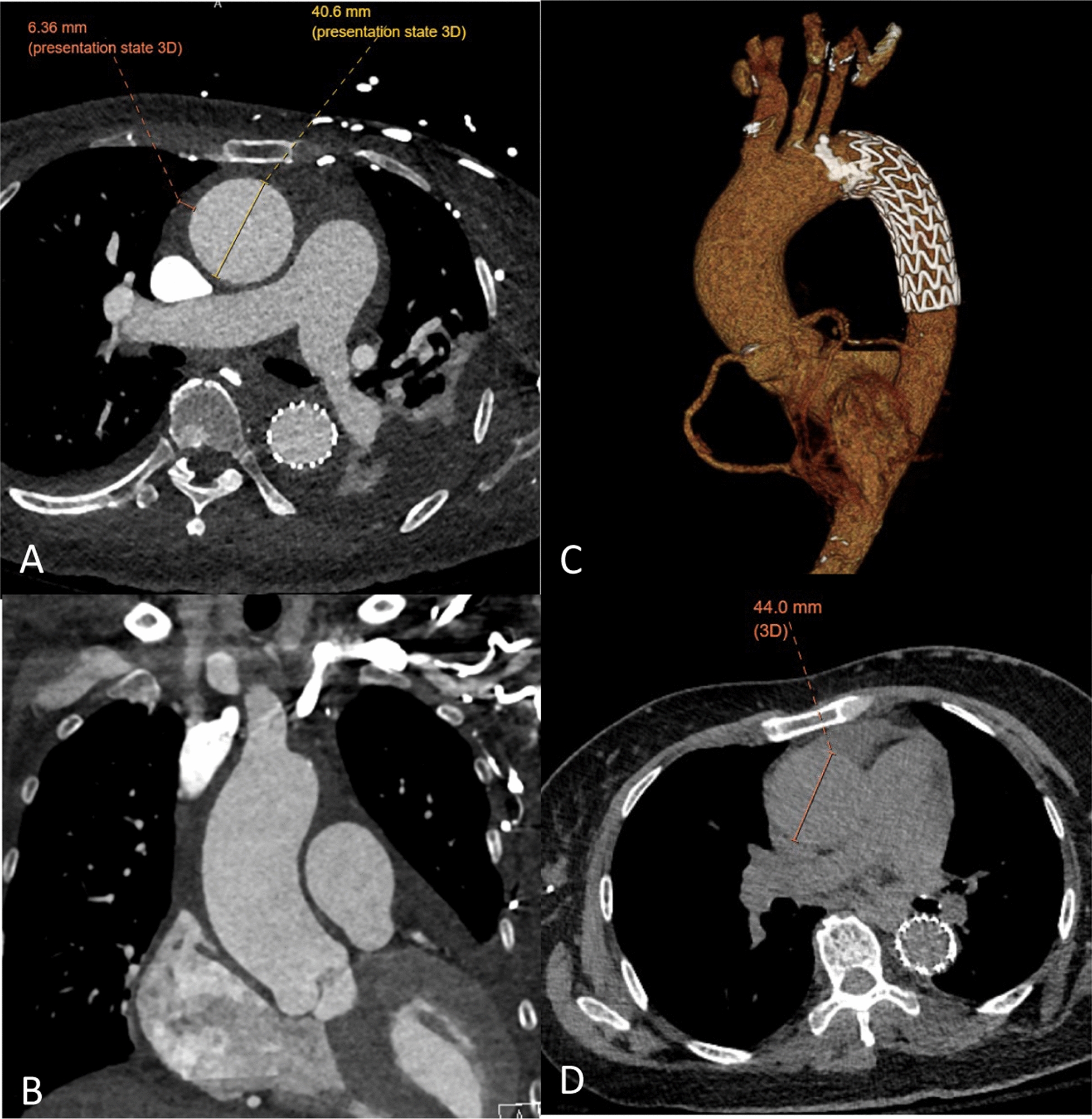
Post-operative Imaging. **A**, **B**, **C** 6 weeks after TEVAR. **D** Non-contrast CT 3 years after TEVAR

The procedure was done under general anesthesia with transesophageal echocardiogram (TEE) assistance. Initial imaging checkpoints included confirming both iliofemoral access and extent and anatomy of disease on both angiogram and intravascular ultrasound (IVUS). The proximal descending aorta was treated with a Gore® c-TAG with active control size 31 × 29 × 100 mm, which was oversized to the true lumen by 6% proximally and 25% distally. The thickness of IMH was stable and had not progressed to dissection on aortogram, TEE and IVUS. Additionally, the pericardial effusion was stable. Her length of stay was 22 days and included drainage of a pleural effusion, cholecystitis treated with antibiotics and antihypertension managements. Post-operative imaging at 1 month demonstrated no progression of disease, stable aortic diameters, stable dissection in the right axillary artery and near complete resolution on the ascending and aortic arch IMH. Radiographic follow up at 3 years, was only done with non-contrast CT but demonstrated a stable TEVAR position and an increase in her ascending aortic diameter from 41 to 44 mm. The remained of the aorta was stable in size (Fig. 3).

## Discussion

The presence of IMH in the ascending aorta is considered an acute aortic syndrome necessitating open surgical repair due to both short-term risks of rupture and long-term risks of aneurysm formation. While some ascending IMH can be seen to regress with medical management, positive remodeling is unpredictable, and it has been seen that 12% will progress to frank type A dissection and 54% will develop aneurysm [1]. Both overall size of the ascending true lumen and thickness of IMH over 11 mm are thought to predict which cases will progress. Surgical therapies for retrograde ascending IMH include ascending replacement, ascending replacement with descending TEVAR or total arch replacement with or without frozen elephant trunk. While surgical outcomes are generally acceptable with 8% operative mortality, [2] complications such as stroke and stent graft migration into the false lumen are reported.

The positive result of TEVAR in retrograde ascending IMH reported here is supported by several case series, one of which contained 65 patients with 94% having a successful result. [3] Another series compared 104 TEVAR cases for retrograde type A IMH to 31 cases undergoing medical management, and demonstrated an improved long-term survival with TEVAR, at 84% at 5 years. [4] How this approach may translate to ascending IMH due to tears in the arch, as endovascular management marches more proximally, remains unclear.

Similarly to TEVAR for acute type B dissection, procedural risks, such as vascular injury or stent graft induced progression of disease, are highest in the freshly injured, actively inflamed aorta. Additionally, the inherently diseased landing zone in cases with retrograde IMH will significantly increase the risk of frank type A dissection. Li et al. suggests the rate of frank RTAD with TEVAR for retrograde ascending IMH is twice that of TEVAR for acute type b dissection, at 4%. [4] However, it remains unclear if intervention can be delayed until the subacute period as it often is for uncomplicated acute type B dissection. This approach relies on high-quality imaging both pre-operatively and intra-operatively. [5, 6] The origin of the IMH must clearly be from an intimal defect in the descending aorta, without flow in the ascending aortic false lumen, for TEVAR alone to be a successful approach. [5] In this case was confirmed on both TEE and IVUS, with imagines compared before and after stent deployment. Additionally, we were prepared to surgically repair her type A if the IMH did progress. Other keys to success include the standard principles for endovascular management of acute type b dissection (minimal oversizing, avoiding ballooning, strict blood pressure management).

By way of a recent case, we have demonstrated that ascending aorta and aortic arch IMH arising from Type B dissection and can be diagnosed by TEE and intravascular ultrasound and treated by TEVAR resulting in a less invasive treatment and possibly a better outcome for patients. [7, 8] We recommend referral to an academic center for Aortic Surgery with TEVAR expertise for patients who present with an ascending aortic or arch intra mural hematoma (IMH).

## Data Availability

No datasets were generated or analysed during the current study.

## References

[1] Evangelista A, Dominguez R, Sebastia C, Salas A, Permanyer-Miralda G, Avegliano G, et al. Long-term follow-up of aortic intramural hematoma: predictors of outcome. Circulation. 2003;108(5):583–9. 10.1161/01.CIR.0000081776.49923.5A. 12874185 10.1161/01.CIR.0000081776.49923.5A

[2] Kim JL, Baiocchi M, Leipzig M, Duda M, Aranda-Michel E, Tognozzi E, et al. Type A intramural hematoma over 21 years: A single center’s experience. JTCVS Open. 2024;26(23):1–18. 10.1016/j.xjon.2024.09.033.PMID:40061525;PMCID:PMC11883695. 10.1016/j.xjon.2024.09.033 PMC11883695 40061525

[3] Li G, Xu X, Li J, Xiong S. Thoracic endovascular aortic repair for retrograde type a aortic intramural hematoma. Front Cardiovasc Med. 2021;30(8):712524. 10.3389/fcvm.2021.712524 PMC8435682 34527712

[4] Li J, Xia L, Ma M, Feng X, Wei X. Outcomes of intramural hematoma involving the ascending aorta and extending into the descending thoracic aorta. J Vasc Surg. 2022;75(1):56-64.e2. 10.1016/j.jvs.2021.07.231. 34481899 10.1016/j.jvs.2021.07.231

[5] Yang KJ, Chi NH, Yu HY, Chen YS, Wang SS, Wu IH. Outcome comparison between open and endovascular aortic repair for retrograde type A intramural hematoma with intimal tear in the descending thoracic aorta: a retrospective observational study. Front Cardiovasc Med. 2021;18(8):755214. 10.3389/fcvm.2021.755214 PMC8558361 34733898

[6] Ryoi O, Lin CH, Chen JM, Hsieh YK, Wang SS, Wu IH. Endovascular repair for retrograde type A intramural haematoma with intimal tear in the descending thoracic aorta. Eur J Vasc Endovasc Surg. 2020;60(3):386–93. 32741679 10.1016/j.ejvs.2020.05.021

[7] Hari Y, Takagi H. Urgent thoracic endovascular aortic repair for type-B_0,_D acute aortic dissection. Ann Vasc Surg. 2024;109:1–8. 10.1016/j.avsg.2024.05.025. 39025222 10.1016/j.avsg.2024.05.025

[8] Wang Y, Song S, Zhou C, Zhu W, Liu J, Shi Q, et al. thoracic endovascular aortic repair for retrograde type a intramural hematoma with intimal disruption in the descending aorta. J Endovasc Ther. 2021;1:15266028211061268. 10.1177/15266028211061268 34852656

